# Drug Loss at Arterial Bends Can Dominate Off-Target Drug Delivery by Paclitaxel-Coated Balloons

**DOI:** 10.3390/pharmaceutics17020197

**Published:** 2025-02-04

**Authors:** Linnea Tscheuschner, Efstathios Stratakos, Marios Kostakis, Miltiadis Gravanis, Michalis Katsimpoulas, Giancarlo Pennati, Fragiska Sigala, Abraham R. Tzafriri

**Affiliations:** 1 Department of Vascular Surgery, National and Kapodistrian University of Athens, 115 27 Athens, Greece; 2 Laboratory of Biological Structure Mechanics, Department of Chemistry, Materials and Chemical Engineering “Giulio Natta”, Politecnico di Milano, 20132 Milan, Italy; 3 Laboratory of Analytical Chemistry, Department of Chemistry, National and Kapodistrian University of Athens, 157 72 Athens, Greece; 4 Department of Interventional Radiology, General Hospital of Athens “G. Gennimatas”, 115 27 Athens, Greece; 5 Experimental Surgical Unit, Biomedical Research Foundation of the Academy of Athens, 115 27 Athens, Greece; 6 Department of Research and Innovation, CBSET Inc., Lexington, MA 02421, USA; rtzafriri@cbset.com

**Keywords:** drug-coated balloons, endovascular drug delivery, peripheral arterial disease, preclinical models, computational simulation

## Abstract

**Background/Objective**: Paclitaxel-coated balloons (PCBs) can deliver efficacious drug concentrations to treated arterial segments but are known to exhibit high tracking losses. We aimed to define the governing factors impacting tracking loss and to contrast its drug distribution consequences with those of PCB inflation at the treatment site. **Methods**: Four naïve and four in-stent restenosis (ISR) porcine superficial femoral arteries (SFA) were treated with PCBs, and plasma samples were collected post-tracking and post-inflation. Animals were sacrificed <1 h post-intervention, and local, upstream, and downstream tissues were collected for paclitaxel quantification. Computationally driven quantitative benchtop-tracking and frictional PCB-sliding experiments modeled paclitaxel loss and delivery to upstream tissue. **Results**: Paclitaxel concentrations in plasma peaked pre-inflation and declined 30-fold immediately post-inflation. Correspondingly, losses of 30% and 1% of nominal PCB load were measured in vitro during, respectively, tracking over single bend and during device insertion. Mean paclitaxel concentrations were equally high at ISR and naïve SFA treatment sites (56,984 vs. 79,837 ng/g, *p* > 0.99) and ranged from 9 to 89 ng/g in tissues downstream of these treatment sites. Sampling of non-target upstream iliac artery tissues revealed paclitaxel concentration of 4351 ± 4084 ng/g. Benchtop sliding of PCB samples onto ex vivo porcine artery samples exhibited efficient, pressure independent frictional paclitaxel transfer (124 µg at 0.05 atm vs 126 µg at 0.1 atm, *p* > 0.99). **Conclusions**: PCB interactions at porcine vessel bends led to premature tracking loss, resulting in peak plasma concentrations exceeding post-inflation concentrations, and delivery to upstream tissue that is plausibly explained as arising from efficient friction-mediated coating transfer.

## 1. Introduction

Peripheral arterial disease (PAD) is a life-threatening disease affecting about 2.5% of the population over 50 and up to 50–60% over 80 [1]. Treatment options for patients with advanced disease manifestations are either open surgery or endovascular revascularization [2]. The last two decades have seen the emergence of drug-coated balloons (DCBs) as a potent endovascular therapy for PAD, demonstrating superior vessel patency and lower incidences of revascularizations, compared to plain balloon angioplasty [3]. DCBs have the potential of complementing or replacing drug-eluting stents for treatment of femoropoptilal lesions due to their often complex nature and the mechanical demands of this highly flexible arterial region. Unlike stents, which serve as a chronic implant scaffold for local drug delivery to the arterial lesion, DCBs are designed to deliver a solid coating that then serves as a mobile drug depot. After DCB retraction the transferred coating dissolves slowly at a prescribed rate, diffuses within the tissue, and binds to its therapeutic target [4]. Dissolution kinetics are dependent on coating morphology, excipient and drug-excipient ratio [5,6,7]. Compared to drug-eluting stents, this approach offers the potential advantages of leaving no foreign material at the lesion site behind, eliminating the risk of late stent thrombosis, stent fracture, and in-stent restenosis [8,9].

Efficacious and safe DCB-coating formulations must balance two opposing design objectives. On the one hand, the drug coating needs to adhere strongly enough to the balloon surface so as to withstand wash-out in blood flow and mechanical interactions with the vascular wall during tracking to the target lesion. On the other hand, a sufficiently high fraction of the coating needs to locally transfer to the target vessel site during very short inflation times (typically 30–180 s). DCB inflation times are limited, since the fully expanded DCB occludes blood flow and therefore stops perfusion of downstream tissue. While in vitro experiments have shown no significant difference in drug transfer between short contact times of 2 s versus longer times of 60 s [10], some recent clinical studies have revealed evidence for improved patient outcomes after treatment of coronary lesions with prolonged (>180 s) DCB inflations [11,12,13], though inflation pressures were not accounted for in these comparisons. Optimizing drug delivery during short inflation times has relied on formulation chemistry, with paclitaxel emerging as the drug of choice in early applications owing to its pharmacology and physicochemical properties [5]. Early in vitro studies with paclitaxel coatings and a range of excipients demonstrated that increasing hydrophobicity of DCB coatings led to a better transfer but simultaneously to more brittle coatings and therefore to higher rates of tracking losses [14,15]. Woolford et al. [6] demonstrated that, with higher hydrophilic excipient ratio, the coating forms a coarser microstructure and is more prone to losses during tracking. This aligns with Kaule et al.’s [15] findings of higher fractions of coating losses of rougher coating, which the authors attributed to frictional interactions during tracking. Such formulation studies have resulted in the development of several clinically approved DCBs, which mostly employ paclitaxel as the released drug and differ in the incorporated dose, the type of excipient employed, the solid morphology of the coating, and also the folding state of the balloon during coating (which influences the coating pattern on the surface of the DCB).

**Figure 1:**
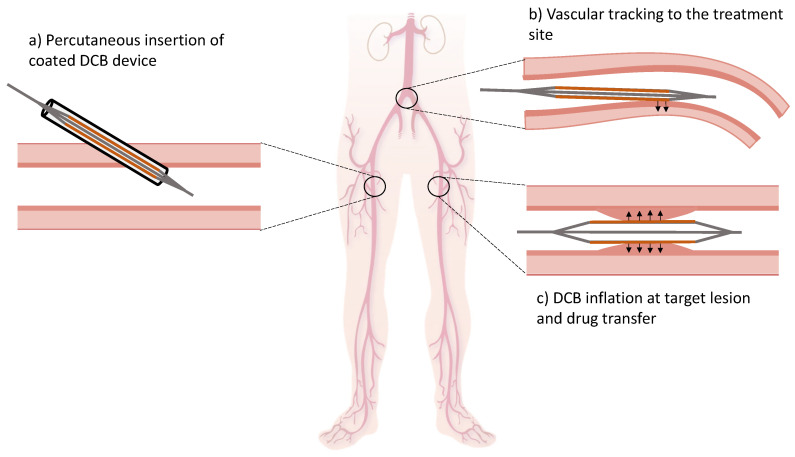
Schematic representation of DCB intervention. (**a**) Firstly, the devices are inserted percutaneously into the arterial system; (**b**) secondly, the devices are advanced through the vascular system upstream to the treatment site under fluoroscopic guidance; (**c**) at the target lesion, the DCB is inflated, and the coated-drug cargo is transferred to the underlying tissue. Modified from InjuryMaps licensed under CC-BY-SA-4.0).

While tremendous effort has gone into the formulation of DCB coatings, the tracking loss remains high even in clinical devices, as a biproduct of the need for efficient local coating transfer to the treated tissue. Drug loss during tracking reduces the available coating for targeted delivery and increases the risk of off-target delivery to systemic plasma and non-target tissue. Optimizing the balance between tracking loss and local targeted delivery remains therefore a key consideration in DCB technology development [16]. To understand the causes for tracking loss and off-target delivery, it is worth reviewing the interventional procedure which begins with DCB insertion into the arterial system through a designated access site (e.g., femoral artery, brachial artery, popliteal artery, etc., [17]) (Figure 1a) followed by its advancement to the treatment site under fluoroscopic guidance (Figure 1b). After angiographic confirmation of DCB positioning at the target lesion, the DCB is inflated for 30–180 s at an assigned pressure that ensures maximal expansion and engagement with the arterial surface [10,18] (Figure 1c).

To date, data on in vivo characterization of tracking loss is sparse, and most current studies focus on analyzing the effects of downstream embolization to assess the safety of the devices. Typically, the DCB is inserted into the arterial system of a rabbit [19] or a swine [20,21,22], inflated in a suitable artery and systemic organs as well as downstream tissue is collected for drug quantification or histopathological assessment [19,21,22,23,24,25]. However, the determinants of such off-target delivery have gone largely understudied. In the current series of non-clinical studies, we undertook the parsing out of the impact of various interventional steps involved in DCB treatments, e.g., insertion, tracking, and local inflation on systemic drug loss in vivo and investigating the location of up- and downstream off-target drug delivery. For this purpose, the drug concentration was sampled in a new spatial and temporal paradigm that included post-tracking/pre-inflation systemic plasma and upstream arterial bends, as fluoroscopy and simulations led us to hypothesize that the latter are loci of elevated interaction and tracking loss.

In vivo observations of the peak systemic drug concentrations immediately post-tracking were complemented by benchtop-tracking experiments to resolve the different contributions of insertion versus tracking along bend to systemic drug loss. The geometries for in vitro tracking were determined by in silico tracking simulations, identifying the bend with highest predicted contact pressure between artery and DCB (upstream arterial bend with frictional contact -UABf). These in vitro tracking experiments confirmed that tracking over a single steep bend can lead to a loss of approximately 30% of the total drug to systemic plasma compared to only 1% during insertion.

Surprisingly, sampling of arterial tissues upstream of the inflation site revealed drug concentrations on par with those achieved at the target site. Benchtop experiments, where DCB samples were pressed onto flat samples of ex vivo arteries with variating contact pressures coupled with longitudinal movement, confirmed efficient drug transfer under conditions mimicking friction during device tracking. These experiments confirmed that “friction-mediated” drug delivery can act as a potent mechanism for drug transfer, revealing a novel drug-delivery mechanism in DCB treatment instead of solely contact-pressure-dependent delivery and explaining efficient in vivo drug delivery to upstream arterial bends sites. Both findings seem to shed light on the often-neglected influence of tracking loss on the overall effectiveness of DCB technology. This study highlights the importance of post-tracking plasmas sampling to assess DCB safety and the urge to protect the DCB coating from frictional loss during device tracking.

## 2. Materials and Methods

### 2.1. Materials

A Super Sheath introducer sheath was purchased from Boston Scientific (Marlborough, MA, USA), PealFlow drug-coated balloons were purchased from L2Mtech (Bonn, Germany), and a 0.035″ guide wire (Radifocus Guide Wire M) was purchased from Terumo Europe N.V., Leuven, Belgium. For the in vitro tracking, PVC tubing (Gloveins) from Global Medikit Limited, Delhi, India, was used.

Methanol, formic acid, and acetonitrile in LC-grade, as well as sodium acetate, were purchased from Merck KGaA, Darmstadt, Germany. Diethylether and sodium cloride were both purchased from Thermo Fisher, Waltham, MA, USA and carbamazipine from Sigma-Aldrich, St. Louis, MO, USA. Pentabarsol was purchased from Dechra Pharmaceuticals, Skipton, England, and polyurethane resin (RESIROCK) from Hobbyland, Legnano, Italy.

The LC-MS/MS system consisted of an ExionLC system and a SCIEX QTRAP 6500+ mass spectrometer, both from AB SCIEX, Framingham, MA, USA, and a Kinetix C18 (100 × 2.1 mm, 2.6 μm) column from Phenomenex, Aschaffenburg, Germany.

### 2.2. Drug-Coated Balloons

All experiments utilized the PearlFlow from L2Mtech (Bonn, Germany), a polyamide balloon that is coated in a folded state with 3 µg/mm^2^ paclitaxel in a 1:9 ratio with butyryl-tri-hexyl citrate (BTHC) excipient.

For the animal study, eight 5 × 60 mm (2962 µg paclitaxel) devices were used; for in vitro tracking, five 2 × 80 mm (1535 µg paclitaxel) devices were used; and for in vitro friction experiments, two 6 × 60 mm (3538 µg paclitaxel) devices were used.

### 2.3. In Vivo Experiments

All animal experiments were carried out at the Biomedical Research Foundation, Academy of Athens (BRFAA- Installation code: EL25BIOexp03), under the approval of the local authorities (License number AΠ: 807280). A unilateral in-stent restenosis (ISR) disease model was created in four male (37–64 kg) Landsrace x Large White crossbreed swine. In each animal, the contralateral naïve SFA was treated as well, using a within-subject control to minimize inter-animal variability. All animals arrived at the unit at least four days prior to surgery to ensure proper acclimatization. Creation of ISR lesions followed the methods of Shammas et al. 2015 [26]. Briefly, animals underwent general anesthesia, and three 30-s plain balloon overstretches (1:1.2–1.3 balloon: artery ratio) in the right superficial femoral artery (SFA) were performed under fluoroscopic guidance, followed by the implantation of two nitinol stents, resulting in a 1 mm vessel oversizing [26]. Twenty-eight days after the stent implantation, animals underwent DCB treatments and angiographic assessment of ISR lesion stenosis degree. Stent placement and naïve DCB treatment sites were selected by lumen diameter while minimizing the presence of branch points and arterial bends. The lesions were pre-dilated with a plain balloon (Mustang, Boston Scientific) inflation to nominal pressure, followed by 3 min DCB (PearlFlow, L2Mtech, Germany) treatment according to the guidelines of the producer (1:1.1–1.2 target balloon: artery ratio). All balloons were inserted through a 6F 25 cm long introducer sheath (Super Sheath—Boston Scientific, Marlborough, MA, USA) placed in the carotid artery and along the 0.035” guide wire. After the ISR lesion treatment, another DCB was advanced into the co-lateral naïve SFA and inflated for 3 min, without prior pre-dilation. The animals were euthanized 30 min after the second DCB inflation by injection of 1 mL/4.5 kg bodyweight pentobarbital sodium 20% (Pentabarsol—Dechra Pharmaceuticals, Skipton, England). Immediately after euthanasia, systemic organs, regional, downstream, and upstream tissue, as well as target arteries were collected for drug quantification with LC-MS/MS.

#### 2.3.1. In Vivo Blood Sampling

Drug loss to systemic plasma during DCB intervention was assessed via sampling of systemic blood through a hemostatic valve at the carotid access site at three different timepoints:

1. Baseline t_0_—at the start of the intervention.
2. Tracking t_1_—immediately after DCB tracking to ISR lesion side (20–45 s).
3. Inflation t_2_—immediately 3 min post DCB inflation at the ISR lesion.

All the plasma samples were collected in K2EDTA Vacutainer tubes and centrifuged to separate plasma from blood components, and all samples were frozen and stored at −80 °C prior to analysis.

Percentage of nominal drug load loss was calculated for each animal, by following standard guidelines of total blood volume equaling 6% of animal weight [27], of which we measured 54% as total plasma volume in the cohort of animals used for these experiments at their day of arrival.

#### 2.3.2. Tissue Collection for Bioanalysis

Immediately after euthanasia, samples of the systemic organs were collected from the lung, liver, and kidney without prior perfusion. At ISR- and naïve-treated-side regional tissues of vastus lateralis (VL), tensor fasciae latae (TFL) were collected, as well as downstream tissue of tibialis anterior (TA) and coronary band (CB). Additionally, for the ISR-side regional tissue, subcutaneous fat was sampled. The prefix “nX” marks samples collected from the treated naïve SFA leg.

Treated ISR SFAs of 4 animals were collected, including about 1 cm of tissue proximal and distal to stent. The 40 mm stented vessel segment was sectioned into 2 equal segments, and the proximal half of the second segment was collected for bioanalysis, while the remaining parts of the SFA was processed for histology. The collected ISR SFA sample was separated into anatomic layers comprised of the area bounded by the lumen and stent (“Intima hyperplasia”), the area bounded by the stent and external elastic lamina (“Intima-media”), and the “Adventitia” layer.

Treated naïve SFAs were collected based on angiographic anatomical markers like branch points and arterial bends, with about 1 cm collected from the middle of the vessel for bioanalysis, while the rest of the tissue was processed for histology.

Total drug contents of treated vessel segments were calculated by measuring vessel weight of treatment length of naïve SFA and multiplied by measured drug concentration in the 1 cm tissue sample.

**Figure 2:**
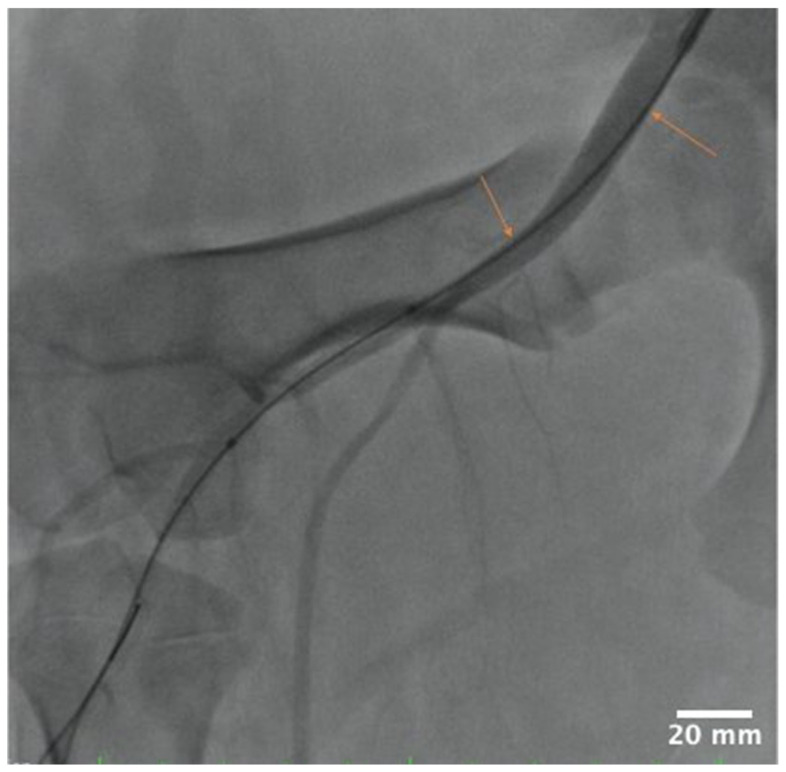
Angiographic image of guide position relative to the lumen of the common iliac artery during stent placement. Orange arrows indicating regions of interaction.

During stent deployment procedures, we observed an interaction of the guide wire with the arterial lumen upstream of the SFA treatment site (Figure 2). To investigate the hypothesis that such interactions can lead to friction-mediated drug delivery by the tracked DCB, we collected ~1 cm long arterial tissues samples of upstream common iliac artery (UABf) for bioanalysis. Carotid artery samples were also collected for bioanalysis to test for potential insertion loss. All samples were flash-frozen and stored at −80 °C until analysis.

### 2.4. Extraction and LC-MS/MS Analysis

Tissue and plasma extraction followed previously published methods [19], with some minor modifications as briefly described. To account for potential drug loss during sample extraction, 50 µL of plasma sample or 50 mg of tissue sample from pigs were spiked with 10 µL of 10 ppb carbamazepine as internal standard. Additionally, 50 µL of sodium acetate buffer (pH 5) was added, for, respectively, plasma or tissue. Tissue samples were diluted with 1 mL of saline and homogenized with a Polytron PT1600 E benchtop homogenizer. All the samples were extracted with 6 mL diethyl ether, centrifuged for 10 min at 5000 rpm, and the upper organic phase was transferred to a clean vial. The organic phase was evaporated under nitrogen flow and samples were reconstituted in 200 µL of mobile phase.

Used DCBs were extracted in an ice bath under sonication (30 min) in 10 mL of methanol spiked with 10 µL of 10 ppb carbamazepine as an internal standard, followed by 1 h of incubation on a rocking table. All the sample vials were sealed with parafilm to avoid potential evaporation.

After extraction, the samples were analyzed with the LC-MS/MS system. The liquid chromatography system consisted of an ExionLC system (AB SCIEX, Framingham, MA, USA), a Kinetex C18 (100 × 2.1 mm, 2.6 μm) column (Phenomenex, Aschaffenburg, Germany), and binary pump. The injection volume of each sample was 10 µL, and the column temperature was 40 °C. Mobile phases consisted of 0.1% formic acid in water (Millipore) (solvent A) and 0.1% formic acid in acetonitrile (solvent B). Chromatography was performed using a gradient program starting with 80% solvent A and decreasing to 0% solvent A in 10 min at a flow rate of 0.2 mL/min. The mass spectrometry system consisted of a SCIEX QTRAP 6500+ mass spectrometer (AB SCIEX, Framingham, MA, USA) with electron spray ionization (Turbo V ion source) in positive ionization mode, working in Multiply Reaction Mode (MRM). Carbamazepine was used as an internal standard to minimize matrix effect and correct signal drift during analysis. The retention times were 4.3 min for paclitaxel and 3.2 min for carbamazepine.

### 2.5. In Silico Tracking Through Blood Vessel and Tubing

**Figure 3:**
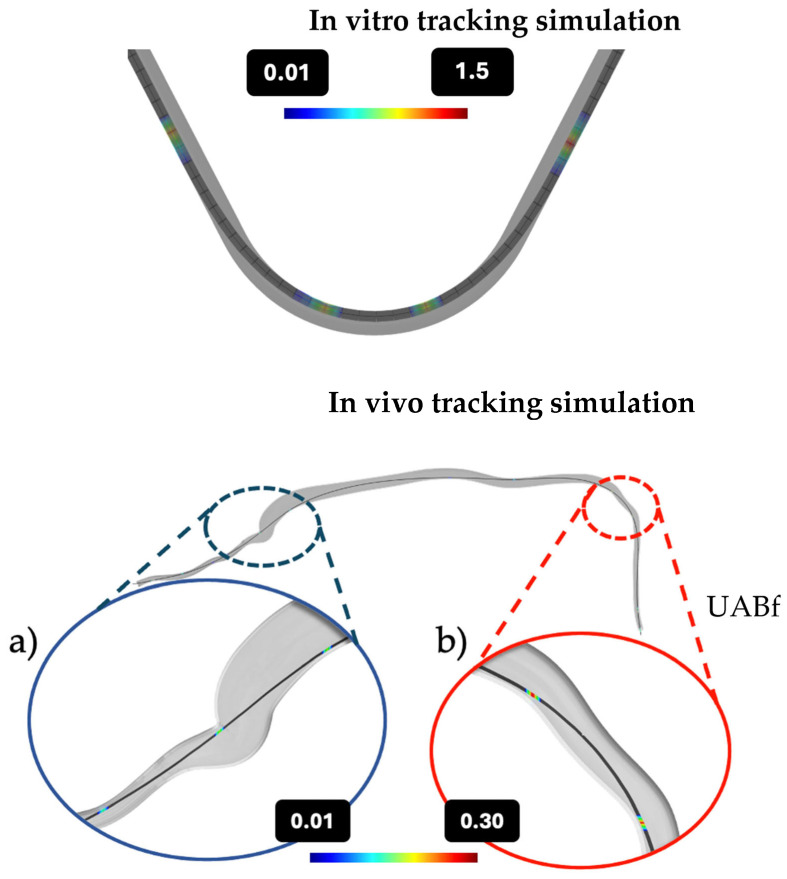
In silico tracking simulation. Upper image: Tubing–DCB interaction during in vitro tracking simulation. Color map showing interaction sides and predicted contact pressure intensity. Lower image: Based on CT data, an idealized arterial pathway from the insertion site (carotid artery) to treatment site (SFA) was reconstructed. Tracking of DCB was simulated along that path, and ”friction hotspots” as well as interfacial contact pressure between balloon catheter and arterial wall were computed. Friction hotspots were detected in the carotid artery, the aortic arch (**a**), and the common iliac artery, later referred to as UABf (**b**), with contact pressures ranging from 0.01 to 0.3 atm.

In vivo DCB–vessel and in vitro DCB–tubing interactions were simulated using Abaqus CAE (Dassault Systems, Paris, France), whereby the DCB and guidewire were modeled as a single entity with a combined equivalent Young’s modulus based on a 3-point bend test (as described in Supplementary Materials). Vessel as well as tubing walls were modeled as rigid and non-deformable surfaces [28]. To simulate in vivo tracking, geometry and dimensions were extracted from published CT data of pigs of similar weight [29,30]. As friction is proportional to normal force, normal forces were calculated during tracking and converted into contact pressures, based on the area of interaction. Computationally predicted areas of increased pressure, implying “friction hotspots”, were identified in the carotid artery, aortic arch (Figure 3a), and in the bend of the common iliac artery (UABf) (Figure 3b).

The predicted contact pressures in these regions ranged from 0.01 to 0.3 atm. However, we anticipated that the rigid wall assumption in the simulation slightly overestimated the actual in vivo contact pressures observed during tracking in the pig vasculature. Based on this, we conducted benchtop-stamping experiments using two extreme compression forces to generate pressures within the 0.01–0.25 atm subrange of predicted contact pressure, aiming to assess the impact of frictional force on acute drug-coating transfer. The numerical replication of the benchtop-stamping experiment demonstrated that applying a force of 0.05 N produced a maximum contact pressure of 0.17 atm, with an average pressure of 0.11 atm. When the force was increased to 0.1 N, the maximum contact pressure reached 0.22 atm, with an average pressure of 0.16 atm (see Supplementary Materials). These two forces were thus selected for the stamping experiments (in vitro friction experiments 2.7).

Based on the CT-extracted vascular geometry, the steepest bend along the tracking path, which was used for benchtop-tracking experiments (2.6), occurs in the common iliac artery and is characterized by a radius of 37 mm, an angle of 25 degrees, and an arc of 8 mm. The DCB–tubing contact length was predicted as 20 mm for in vitro tracking, while the DCB–vessel contact length was predicted to be 36 mm for in vivo tracking.

### 2.6. In Vitro Tracking in Tubing

**Figure 4:**
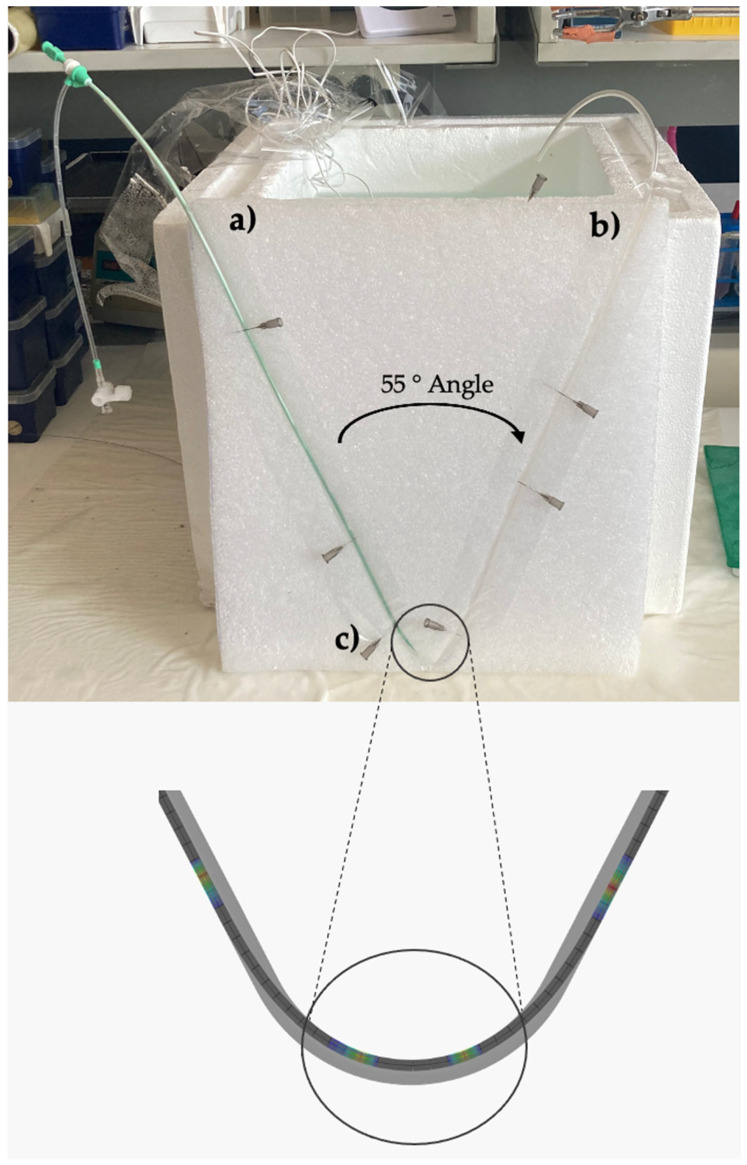
Benchtop set-up for in vitro tracking: (**a**) introducer sheath, (**b**) PVC tubing, and (**c**) bend with 37 mm diameter predicted as bend with highest contact pressure values in simulations (UABf).

For the in vitro tracking experiments, the in vivo tracking path was simulated using a 2.8 mm diameter medical grade PVC tubing (Figure 4a) of 95 cm length (matching the average tracking distance in in vivo experiments). For DCB insertion, the same 6F 25 cm introducer sheath (Super Sheath, Boston Scientific, USA) as employed in the in vivo experiments was inserted into the PVC tubing (Figure 4b). The tubing was fixed in a styrofoam plate to create a bend in the middle of the tracking path with the smallest diameter of about 37 mm, which resulted in an angle of 55°, deduced from simulations as the bend with highest contact pressure during tracking (UABf) (Figure 4b). Prior to DCB insertion, the introducer sheath and tubing were filled with 6 ml 37 °C warm porcine plasma and immersed in a 37 °C warm water bath for the full length of the experiment. Plasma was collected from two animals of the in vivo study at a stent placement as described in 2.3.1 “In vivo blood collection” and stored at −80 °C until usage. For the experiment, the protective sheath of a 2/80/150 PearlFlow DCB was carefully removed and stored for quantification. The DCB was inserted into the benchtop set-up and pushed until the end of the introducer sheath, just before the bend. A 1 mL plasma sample was collected (“Post-Insertion Plasma”), and the subtracted plasma volume was gently replaced with fresh plasma. Then, the DCB was pushed over the bend until the end of the tracking path; plasma in tubing (“Post-tracking Plasma”) and post-tracking DCB (“Remain on DCB”) were collected for quantification. The introducer sheath and tubing were flushed with 10 mL of methanol to collect the remaining paclitaxel on the tubing wall (“MeOH wash tubing”). The procedure was repeated 5 times each with a new DCB and tubing.

### 2.7. In Vitro Friction Experiments

**Figure 5:**
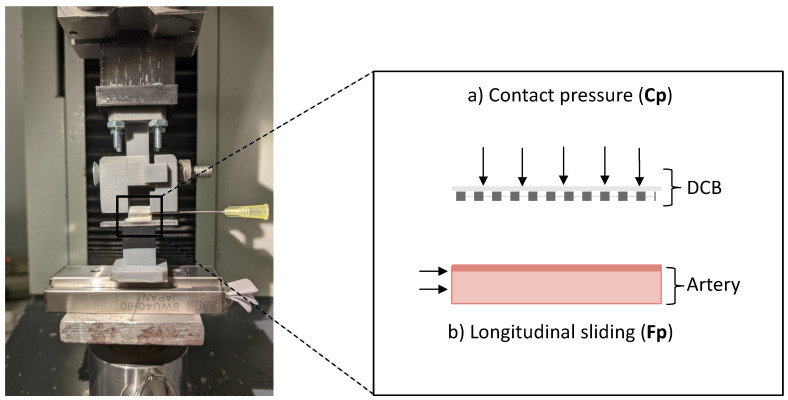
Controlled coating transfer to ex vivo artery samples. Left: a uni-axial tensile test machine was used to press the DCB specimen onto a porcine ex vivo artery (**a**) at a specified target contact pressure (Cp). The artery was fixed on a uni-directional movable stage to be able to mimic the friction condition (Contact pressure + longitudinal movement) (**b**).

In vitro friction experiments utilized the benchtop set-up of Stratakos et al. [10] with the modification of moving the lower part, that contains the ex vivo porcine arterial specimen, to a single-directional movable stage (Figure 5a). The arterial samples were collected from the aortic arch and descending aorta of two swine with approximately the same size as the animals used in the in vivo study, kindly provided by a local abattoir, and were cleaned from surrounding fat and fascia. Arterial sections with approximately the same thickness of 2 (± 0.4) mm were cut into specimens with a width of 5 mm and a length of 10 mm. Prior to experiments, all arterial samples were allowed to equilibrate for at least 1–4 h in 0.9% NaCl solution at 37 °C until usage. DCB samples [10], consisting of cylindrical slices of about 1 cm length, were prepared by infusing fast-curing polyurethane resin (RESIROCK, Hobbyland, Legnano, Italy) into balloon lumen and allowing it to harden for 25 min.

Benchtop experiments simulated two in vivo scenarios using resin-infused DCB samples: (condition i) frictional coating transfer upstream of the treatment site and (condition ii) subsequent pressure-driven transfer of the residual coating at the inflation site. The frictional coating transfer to an upstream artery wall was simulated by applying low pressures (Figure 5a), followed by a longitudinal movement of the arterial specimen (Figure 5b). Experiments were conducted by pressing the specimen at forces compatible with the minimal and maximal contact pressures deducted from tracking simulations (as described in Section 2.4), respectively Fp_L_ = 0.11 atm and Fp_H_ = 0.16 atm). All experimental conditions were replicated in quadruples. Post-frictional coating was extracted from arteries with methanol and analyzed with LC-MS/MS (using the methods described in Section 2.3).

In a second set of experiments, the DCB specimens underwent a primary friction (Fp) interaction as described above, followed by a subsequent inflation (Cp) interaction, for which the DCB specimen was pressed with higher forces onto another arterial sample (Figure 5a), without sliding (mimicking on-target DCB inflation). Pressure ranges for the inflation interaction (Cp) were deducted from previous in silico inflation simulations [10]. Each step was performed on a separate artery, to simulate friction during in vivo tracking across arterial bends, followed by DCB inflation at the treatment site. Arterial specimens were collected, extracted with methanol, and analyzed for quantity of transferred drug with LC-MS/MS (using the methods described in Section 2.4).

### 2.8. Statistics

Results for continuous variables are expressed as mean ± standard deviation. For in vivo measurements, a Friedman test was used with a pairwise Dunn’s post hoc analysis. For in vitro friction experiments, Kruskal–Wallis with a pairwise Wilcoxon rank sum test post hoc analysis was used. A *p* value of < 0.05 was considered statistically significant, and RStudio (2020, Boston, MA, USA) was used for statistical analysis.

## 3. Results

### 3.1. In Vivo Plasma Kinetics

For validation of analytical methods, four animal samples were spiked with 10 ppb and 50 ppb of paclitaxel each, extracted, and measured. Measurements resulted in a relative standard deviation (RSD) of 14.36% and 9.33% for samples spiked with 10 ppb and 50 ppb paclitaxel, respectively.

**Figure 6:**
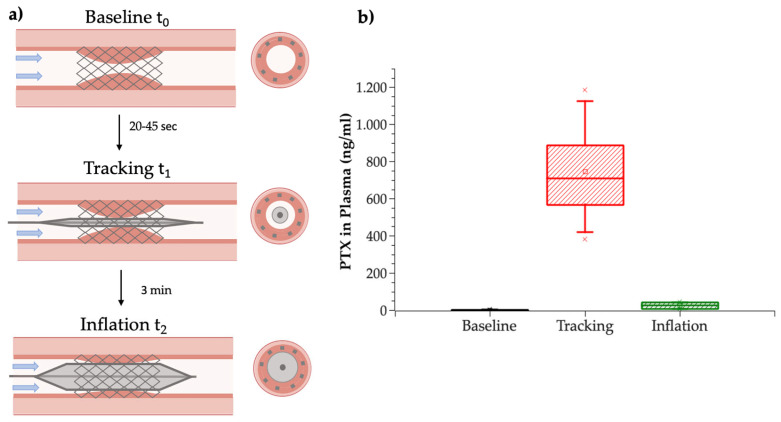
Plasma concentration peaks with tracking loss. (**a**) Schematic representation of in vivo experimental protocol. Systemic plasma samples for drug quantification were collected at the following time points: Baseline t_0_—at the beginning of the intervention; Tracking t_1_—immediately after DCB tracking to the ISR lesion side; Inflation t_2_—immediately after the 3 min DCB inflation at the ISR lesion. (**b**) Paclitaxel concentration in circulating plasma measured at three time points. Highest paclitaxel levels (ng/ml) in circulating plasma were measured during device tracking, followed by a 30-fold decrease after inflation. Matrix effect for all samples were low (Baseline).

Systemic paclitaxel concentration in plasma during different interventional steps (Figure 6a) was highest after tracking (746.9 ± 336.3 ng/mL), followed by a 30-fold decrease to 24.6 ± 20.2 ng/mL immediately after DCB inflation at the ISR lesion (Figure 6b) (*p* = 0.023). Drug concentrations in baseline (t_0_) samples, collected to quantify matrix effects of plasma on paclitaxel concentration measurements, were confirmed to be negligibly low for all measured samples.

The total circulating paclitaxel, as a percentage of the nominal drug load, was 44.5 ± 11.1% during tracking and 1.5 ± 1.3% immediately post-inflation. At euthanasia, 2.4 ± 4.5% of the initial drug load was found in systemic plasma.

### 3.2. In Vivo Drug Distribution into Local and Off-Target Tissues

**Figure 7:**
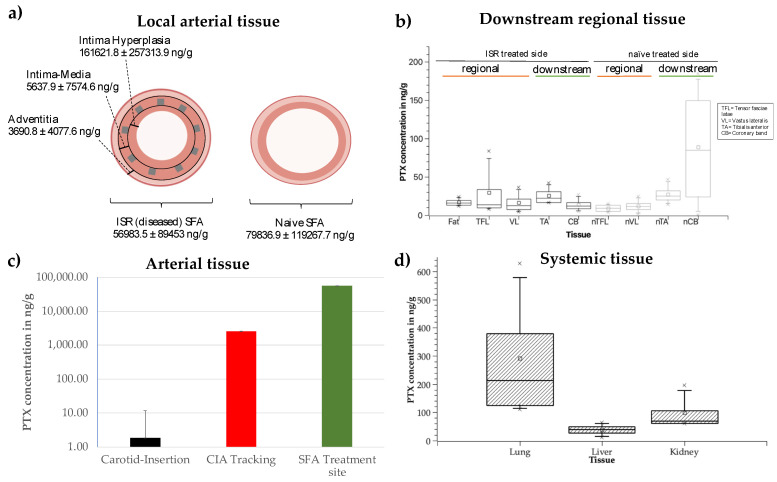
Drug concentrations in local, regional, and systemic organ tissues. (**a**) Local arterial paclitaxel concentration in different layers of treated ISR vessel segments (left) and treated naïve vessel segments (right). No statistically significant difference in total drug concentration in ISR and naïve vessel segments. (**b**) Paclitaxel concentration in regional downstream tissue, left-side ISR SFA treated leg (black), and right-side naïve SFA treated leg (gray). (**c**) Paclitaxel concentration in different arterial segments: black: carotid artery at the insertion side, red: common iliac artery (CIA) on tracking route, corresponding to UABf, green: SFA at inflation site. (**d**) Paclitaxel concentration in systemic tissue shows highest concentration in lung tissue.

Just prior to DCB treatments, the ISR lesion average diameter ranged from 4 to 4.5 mm, and the minimal lumen diameters ranged from 2.8 to 4 mm. Angiography post-balloon inflations confirmed that the balloon/artery ratios were within the target of 1.1−1.26 at all treatment sites. No adverse effects were detected throughout the duration of the animal study, and therefore, no animal samples were excluded from the study. Residual drug on DCBs used at ISR and naïve side showed no statistically significant difference (6.1 ± 2% and 4.2 ± 1.2%) (*p* > 0.99). Treated ISR lesions exhibited a high paclitaxel concentration, with a maximal concentration achieved in the intima hyperplasia layer (161,621.8 ± 257,313.9 ng/g), followed by 34-fold and 63-fold lower concentrations in, respectively, the intima–media layer (5637.9 ± 7574.6 ng/g) and adventitial layers (3690.8 ± 4077.8 ng/g) (Figure 7a). The statistical analysis showed significant dependence of drug concentration and penetration depth between intimal hyperplasia and adventitia layer (*p* = 0.042). Contra-lateral naïve treated SFA showed comparable mean drug concentrations of 79,836.9 ± 119,267.7 ng/g (*p* > 0.99). The average total drug content of naïve SFA was measured as 26,523.2 ± 34,314.3 ng, accounting for 0.9 ± 1.2% of the nominal drug load.

Drug concentrations in regional tissues surrounding the treated vessel (subcutaneous fat, tensor fasciae latae (TFL), vastus lateralis (VL)) and downstream of it (anterior tibialis (TA), coronary band (CB)) for ISR treated vessel and naïve vessel (indicated with “nX”) ranged from 9.3 ± 5.1 ng/g (nTFL) up to 88.7 ± 86.6 ng/g (nCB) (Figure 7b). No statistically significant difference was detected between concentrations in both legs or between regional and downstream tissue (*p* > 0.99).

Sampling of UABf revealed a concentration of 4350.8 ± 4083.6 ng/g paclitaxel, which per limb corresponds to 5.4–46.5% (19.5 ± 17.2%) of paclitaxel concentration in the corresponding SFA. In the carotid artery, concentrations of 1.9 ± 1.5 ng/g were measured (Figure 7c).

Compared to average regional tissue concentration, higher drug concentrations were found in samples of lung tissue (277.2 ± 228.1 ng/g), kidney tissue (92.8 ± 61.2 ng/g), and liver tissue (35.3 ± 20.1 ng/g) (Figure 7d). Drug concentrations in lung tissue were significantly elevated compared to liver tissue (*p* = 0.02).

### 3.3. In Vitro Simulation of Insertion Loss, Tracking Loss and Off-Target Wall Delivery

**Figure 8:**
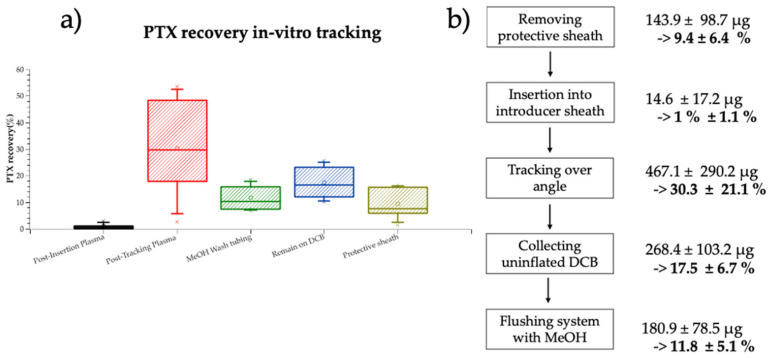
In vitro plasma distribution in benchtop-tracking experiments. (**a**) Measured paclitaxel loss in each procedural step and the associated percentage of nominal drug coating. (**b**) Paclitaxel recovery from in vitro tracking. Highest percentage of drug was recovered from plasma post-tracking over angle.

Benchtop-tracking experiments revealed peak drug loss during tracking over the bend (467.1 ± 290.1 µg; 30.3 ± 21.1% of initial drug load), while insertion only led to a drug loss of 1 ± 1.1% (14.6 ± 17.2 µg). Flushing of the tubing with methanol recovered 11.8 ± 5.1% (180.9 ± 78.5 µg) of the nominal load. Quantification of the protective sheath revealed 143.9 ± 98.7 µg of the drug (9.4 ± 6.4% of nominal DCB drug loading). Post-tracking DCB had 268.4 ± 103.2 µg (17.5 ± 6.7%) of the residual paclitaxel (Figure 8a,b).

### 3.4. In Vitro Frictional Drug Delivery

**Figure 9:**
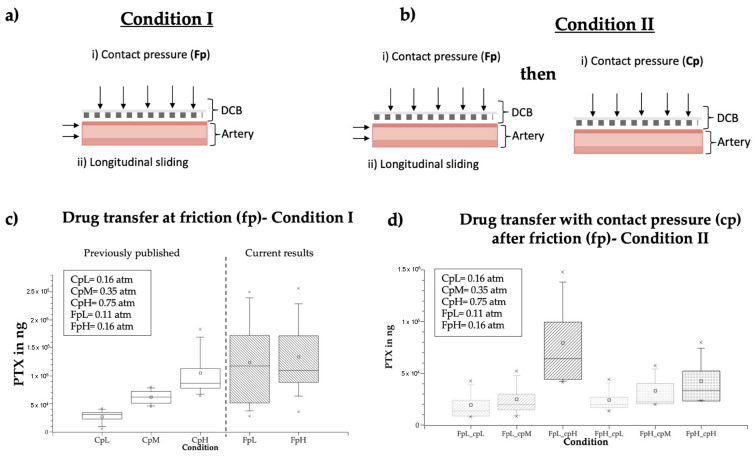
In vitro drug transfer in benchtop friction experiments: (**a**) experiments were performed under two conditions: condition I—mimicking different fiction with different contact pressures, or (**b**) condition II—with the same DCB specimen, an initial contact pressure was performed, followed by a friction condition with the same balloon specimen on a new artery (mimicking tracking followed by inflation). (**c**) Paclitaxel transfer on arterial specimens at condition I—for comparison, previously published [10] average Cp values are depicted on the left. (**d**) Paclitaxel transfer on arterial specimens at condition II.

To assess whether in silico predicted “friction hotspots” (Figure 3) might explain our findings of high drug delivery to UBAf (Figure 7c), we utilized a benchtop set-up to compare “frictional transfer” (Figure 9a) to subsequent “contact pressure driven transfer” (Figure 9b) at representative normal forces derived from simulations.

A first set of experiments quantified drug delivery following longitudinal frictional interaction with ex vivo artery strips at pressures representative of the maximal (Fp_H_—0.16 atm) and minimal (Fp_L_—0.11 atm) in vivo pressures experienced during catheter–vessel upstream of the treatment site (Figure 9a). Bioanalysis of tissue samples revealed near-constant high drug transfer at the low and high pressures, respectively, 124 ± 71.5 µg and 125.8 ± 55.1 µg (Figure 9c).

In a secondary approach, we performed contact pressure stamping after friction with the same balloon specimen to assess the influence of pre-term frictional interaction on drug transfer during inflation, mimicking in vivo tracking with subsequent inflation with the same device (Figure 9b).

Bioanalysis of tissue samples revealed that contact-pressure-mediated delivery scaled inversely with pre-term frictional transfer. Specifically, the lowest transfer was measured for samples that experienced the lowest contact pressure after the friction interaction (Fp_L_ cp_L_: 19.3 ± 16.1 µg and Fp_H__cp_L_: 24.2 ± 13.6 µg, respectively) followed by both conditions with medium contact pressure, regardless of the initial friction pressure (Fp_L__cp_M_: 24.8 ± 19 µg and Fp_H__cp_M_: 33.2 ± 21.1 µg, respectively). For both friction conditions, the highest transfer was measured at the highest contact pressure, yet a higher initial friction pressure led to compromised drug transfer in secondary stamping (Fp_L__cp_H_: 79.3 ± 49.3 µg; Fp_H__cp_H_: 42.4 ± 26.4 µg) (Figure 9d).

## 4. Discussion

Angioplasty balloons with slowly dissolving but transferable paclitaxel formulations have been proven to be efficacious in treating coronary and peripheral artery disease and are now being explored for treatment of strictures in airways [31,32], gastrointestinal [33,34], and urinary tracts [35]. Transferable coatings designed for local delivery at the DCB inflation sites are known to also be transferred nonspecifically prior to DCB inflation. This has inspired the intensive nonclinical study of coating embolization downstream of DCB inflation, owing to its potential safety implications [36]. By contrast, little attention has been paid to drug distribution arising from premature coating transfer upstream of DCB treatment sites. To fill this void, the current set of in vivo, in silico, and in vitro studies were designed to assess the mechanisms driving premature coating loss prior to DCB inflation and to identify the contributions of tracking loss to drug distribution into plasma and delivery to arterial tissue upstream of the DCB inflation site.

As we proceed to detail below, through the coupling of a novel in vivo sampling paradigm for drug in plasma and tissue with dedicated computational modeling and benchtop experiments, we can now begin to provide a coherent picture of device vessel interactions, drug release, and distribution kinetics during the ordered sequence of interventional steps, from insertion through tracking and inflation.

### 4.1. Insertion

Every DCB intervention commences with device unpacking and removal of the protective sheath. Quantification of residual drug on the protective sheath in our in vitro tracking experiment showed already a reduction in available drug coating of 9.4 ± 6.4% (Figure 8). Unsheathing is then followed by device insertion into the hemostatic valve of an introducer sheath, which for the DCB we evaluated, resulted in a loss of 1 ± 1.1% nominal drug load in vitro (Figure 8). Both in vitro results align with published coating loss estimates during removal of protective sheath and insertion optically for three commercial DCBs by Faenger et al. [37]. They found 0.4−12% coating loss during unsheathing, which aligns with our slightly higher measurement of 1.5–16.3%, while they could not detect loss during insertion. We measured a small fraction of 1 ± 1.1% loss during insertion using LC-MS/MS, which has a higher sensitivity for small quantities of coating loss compared to optical analysis.

### 4.2. Tracking

#### 4.2.1. Coating Loss to Plasma Is Dominated by Tracking Loss at Bends

Post-insertion, the device is tracked to the target lesion, thereby subjecting the DCB surface coating to shear stresses in flowing blood, mechanical stresses due to balloon deformation, and frictional forces at bends and DCB–vessel interactions sites along tracking path. Such forces give rise to coating loss to plasma as well as to some coating transfer to the tissue interaction sites.

Sampling of drug in plasma immediately after key in vivo interventional steps revealed that plasma concentration peaked prior to DCB inflation and correlated with tracking loss. Paclitaxel concentration in systemic plasma immediately after tracking was at 746.9 ± 336.3 ng/mL and dropped about 30-fold to 24.6 ± 20.2 ng/mL directly after inflation (*p* = 0.023). As coating is released in a solid form that slowly dissolves over hours to days, it is safe to estimate the peak drug concentration as the ratio of released solid coating during tracking and plasma volume [4]. Doing so implies a loss of 44.5 ± 11.1% of the nominal drug load during tracking. In the literature, plasma sampling for drug loss typically starts about 1−3 min after DCB inflation [23,38,39] (Supplementary Table S1), so there are no direct correlates for our pre-inflation findings. Reported maximal plasma concentrations in animals and humans range from 1.4 to 54.4 ng/mL [23,40,41], similar to our finding immediately post-inflation. Our findings imply that safety testing and drug-loss evaluation studies for DCBs should include earlier timepoints, especially post-tracking and pre-inflation sampling, to adequately evaluate systemic loss of paclitaxel.

To parse the contributions of insertion and tracking over bends to premature coating loss prior to DCB inflation, we examined these issues under more controlled conditions, using a dedicated in vitro tracking model. The specially designed benchtop tracking model evaluated DCB insertion into the same introducer sheath used in the animal study followed by tracking over the steepest bend on the tracking path (“UABf”). This segment, characterized by the smallest radius of curvature along the tracking path from the carotid artery to the SFA, was selected based on an in silico simulation predicting the highest contact pressure during tracking (Figure 3). Sampling of plasma through this benchtop set-up demonstrated that tracking over a single steep bend can lead to a 30.3 ± 21.1% nominal drug load loss to surrounding plasma, underlining tracking over bends as the primary determinant of coating loss to plasma in the device tested here. The relatively high variance in the in vitro tracking samples (of 21%) is at least partially explained by a cumulative effect of variable coating loss onto the protective sheath and during manual handling. Comparing our results to previously published literature remains challenging due to high sensitivity of tracking loss to device-specific coating and treatment-specific tracking geometries. After passage through a blood-filled guide catheter followed by a 1 min stirring in blood, Kelsch et al. [42] detected a drug loss of 26 ± 3% and 36 ± 11% for urea- and iopromide-based DCBs, respectively. While Kaule et al. [15] reported a drug loss of 21–39% after the passage of DCBs with different formulations, through an empty guide catheter along a torturous path. Our in vivo measurement of 44.5 ± 11.1% of nominal drug load is only slightly higher, despite tracking along multiple bends and additional exposure to dynamic blood flow.

#### 4.2.2. Coating Transfer to Arterial Tissues During Tracking

Confirmation of the catheter–vessel interaction upstream of the target lesion at in silico predicted bends (Figure 2) also leads us to hypothesize friction-mediated arterial drug transfer at these sites. Tissue sampling at these in silico predicted upstream friction sites confirmed the presence of substantial drug concentration, with paclitaxel concentrations ranging between 1565.8 and 10,286.6 ng/g (4350.8 ± 4083.6 ng/g) in samples of UABf, amounting to 5.4–46.5% of the corresponding concentrations at the SFA treatment sites. In contrast, the paclitaxel levels measured in the carotid artery were considerably lower at 1.9 ± 1.5 ng/g, demonstrating the protective effect of the introducer sheath against non-targeted upstream arterial delivery.

While our in vivo results do not allow for direct parsing of the percentage of frictionally transferred upstream arterial drug delivery, an approximation can be deduced from the mass balance of the above-presented results. Namely: 9.4 ± 6.4% of coating loss is associated with removal of protective sheath, 1 ± 1.1% is lost in insertion, 45.5 ± 11.1% is lost to systemic plasma during tracking, and 0.9 ± 1.2% is delivered to the target, while 4.2 ± 1.2% remains on the DCB, leaving 39 ± 11.1% of the nominal drug load for frictional upstream delivery.

Recovery of methanol wash-out of tubing after in vitro tracking was consistent with 11.8 ± 5.1% of the nominal drug load being frictionally transferred to the PVC surfaces. In silico simulation of in vitro tracking predicted a contact length of 20 mm for the 80 mm balloon, while for in vivo tracking, a contact length of 36 mm was predicted. Simple scaling of the in vitro delivery by the ratio of predicted in vivo and in vitro DCB contact lengths (36/20) estimates that 47.2% of the nominal drug load is frictionally transferred during in vivo tracking. Notably, this estimate is in concordance with in vivo mass balance estimates.

### 4.3. Inflation

Following DCB tracking and angiographic confirmation of its correct placement at the target lesion, the DCB is inflated with the aim of delivering its coating cargo to the vessel lumen through a contact-dependent mechanism (Figure 1) [43]. Published benchtop experiments with the PearlFlow DCB have demonstrated a positive correlation of coating transfer with contact pressure [36]. Given the dependence of inflation-driven coating transfer on DCB–vessel contact pressure, it is natural to ask whether coating transfer and downstream embolization are dependent on lesion morphology and stiffness. To address this question, we applied the same inflation protocol in ISR and naïve SFA sites, hypothesizing that the differences in intimal composition and the presence of an underlying non-compliant stent in ISR lesions might result in differential drug deposition and drug concentrations in regional and downstream tissue. However, no statistical difference was detected between delivery to both vessels as well as residual drug on the DCBs. Thus, for the DCB we evaluated, neither the differences between native intima and injury-induced neointima nor decrease in compliance [44] due to the embedment of stiff metallic stent within the lesion affected inflation-driven delivery [37]. Paclitaxel concentrations at the ISR arterial treatment sites were measured 56983.5 ± 89453 ng/g, similar to published reports on other devices [22,23,38,45]. Comparing to previously published PCB studies [38,39,41,45], it becomes apparent that measurements of target drug concentrations are often accompanied by high variances (Supplementary Table S1). Since most clinically used DCB coatings, including the one studied here, do not utilize a protective layer around their active drug coating, they are vulnerable to premature drug release due to straining and shearing during tracking, as demonstrated in the presented work. The extent of premature coating loss is dependent on the specific geometry of the tracking vasculature and operator-specific interventional technique. Additionally, variance in diameter along the treated vessel segment can lead to variations in contact forces between DCB and vessel wall [18]. This could potentially affect the degree to which coating embeds into the arterial tissue and therefore its susceptibility to wash-out following DCB retraction [46]. Our sampling of concentric tissue layers in the ISR segments provided direct evidence for coating embedment and interestingly for an intima to adventitia concentration gradient within the first hour post-treatment. Thus, neointimal hyperplasia limits the penetration of coating particles, but not entirely. Similar findings have been reported after DCB treatment of pre-injured porcine coronary arteries wherein paclitaxel distribution was resolved into vessel surface and artery wall components both for crystalline and amorphous coating after 1 h of treatment [47].

Since drug coating experiences substantial mechanical strains during balloon inflation, one could hypothesize a spike in systemic plasma concentration immediately post-inflation, which potentially could contribute to accumulation in coating particles in the regional and downstream tissue of the inflation site. However, our in vivo plasma sampling does not support this hypothesis for the PearlFlow DCB, estimating only 1.5 ± 1.3% of the nominal drug load in systemic plasma immediately post-inflation.

Measured drug concentrations in regional and downstream tissue within 1 h of DCB treatment were low (typically < 50 ng/g) relative to published reports of 91.5−1150 ng/g for other paclitaxel-coated balloons in healthy arteries [21,22] (Supplementary Table S1). High variability in reported downstream tissue concentration for various DCB devices underlines the absence of a class effect of DCBs, which underscores the importance of performing safety and efficacy measurements independently for each device [48].

Additionally, low systemic paclitaxel levels immediately post-inflation and increased tissue concentrations at later time points [22] might indicate that the accumulation of drug in regional and downstream tissue does not mainly arise from particle loss during inflation but rather from detachment and wash-out of already-delivered particles from target lesion post-intervention.

### 4.4. Frictional Versus Inflation Driven Coating Delivery

Kaule et al. [15] reported, in a published in vitro study, the importance of coating roughness on tracking loss, highlighting the potential key role of frictional delivery during tracking loss. We therefore hypothesized that our in vivo findings of drug delivery to UABf tissue are predominantly driven by frictional coating transfer resulting from the combination of a radial contact force and longitudinal sliding.

**Figure 10:**
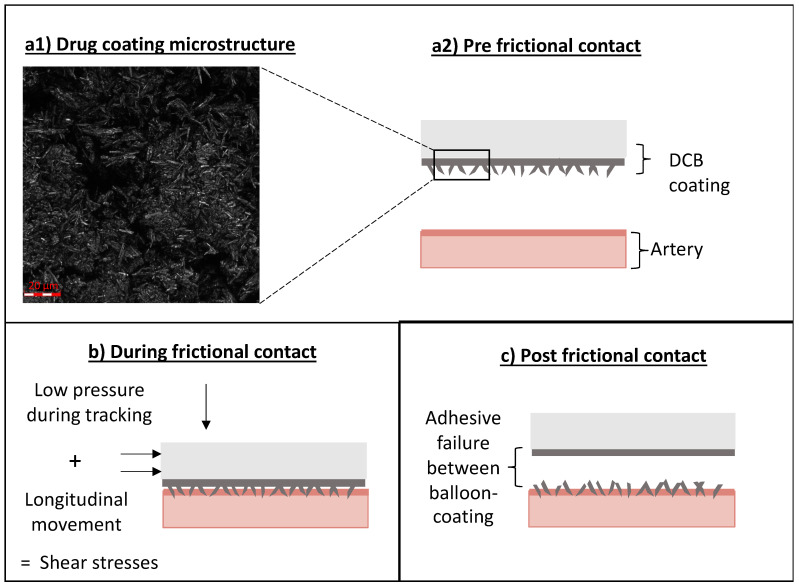
Schematic representation of mechanisms of frictional transfer. (**a1**) Confocal laser microscope image of PearlFlow coating reveals microneedle-like microstructure (100×). (**a2**) Pre-frictional contact: The device is coated with a rough coating of microneedle-like particles, before interacting with the artery. (**b**) During frictional contact: The rough surface coating of the tracked DCB experiences shear forces due to a combination of a low vertical pressure exerted onto the flat arterial tissue sample and the concomitant longitudinal motion. (**c**) Post-frictional contact: The shear stresses during frictional contact lead to an adhesive failure between the balloon membrane and coating layer, leading to an efficient transfer of drug coating to the upstream arterial tissue.

Herein, we utilized a benchtop model to controllably apply contact pressures representative of interactions at bends and the longitudinal motion to DCB specimens (Figure 5). The applied contact pressure ranges applied in these in vitro experiments were derived from in silico simulations of DCB tracking in in vivo extracted vascular geometry. Benchtop experiments showed pressure-independent high drug transfer during friction conditions (Figure 9a,c). At a material level, this implies that the shear forces exerted on the coating exceed coating–balloon adhesion or coating–coating cohesion at multilayered coating areas [16] already at 0.05 atm (Figure 10).

Moreover, benchtop simulation of DCB tracking followed by its inflation revealed higher delivery during the preliminary frictional step (Figure 9b,d). Thus, even low-pressure frictional interaction of the DCB upstream of the inflation site is predicted to significantly deplete the amount of coating available for inflation driven transfer at the target tissue site.

The benchtop set-up we employed here to study the frictional drug transfer to tissue samples had previously been employed by us to quantify contact-pressure-driven drug transfer from the same DCB type in the absence of friction [10]. In that study, we reported drug transfers of 27.2 ± 15.4 µg, 62.1 ± 15.7 µg, and 105 ± 53 µg at contact pressures representative of DCB inflation, respectively, 0.16, 0.35, and 0.75 atm. These contact pressures are all equal or higher than the contact pressure employed in the frictional experiment. Nevertheless, frictional delivery was significantly higher, even when effected at 0.11 atm: 125.8 ± 55.1 µg (*p* = 0.001). Comparing the results from both sets of experiments indicates that frictional drug transfer can exceed inflation driven transfer.

To understand the mechanism of frictional drug transfer on a material level, it is important to recall that the studied coating comprises multiple layers of microneedle-like structure, as preliminary confocal laser microscopy images have indicated (Figure 10a1). The high drug concentrations in post-tracking plasma sampling suggest that some of the coating layers detach under tracking conditions. The peaking of plasma concentrations prior to DCB inflation indicate that a sizable fraction of coating, that is detaching during tracking, is systemically distributed. Surprisingly, the high drug concentrations in UABf samples imply that a substantial fraction of detached coating is transferred to friction-subjacent tissue and retained there despite hemodynamic forces (Figure 7c). During tracking at vascular bends, the DCB coating experiences shearing due to a combination of low pressure pressing of the balloon onto the arterial wall and the concomitant longitudinal motion (Figure 10b). As our in vitro frictional experiments revealed, in vivo relevant shear stresses are strong enough to create adhesive failure between the balloon membrane and the coating (Figure 10c), resulting in efficient transfer of detached coating particles to the underlying tissue.

The finding that frictional transfer can exceed inflation driven transfer has a range of procedural implications. On the one hand, it suggests the exploration of frictional coating transfer as a novel mechanism for effective local drug delivery from DCB via low-pressure sliding motion along the target lesion, primarily via axial rotation. This is of relevance to non-vascular delivery and is especially attractive in the setting of vascular delivery for lesions, where high-pressure inflation is contraindicated.

On the second hand, these findings have implications for the standard usage of DCBs for inflation-driven coating transfer, wherein tracking loss limits the available drug for local delivery at the target lesion. In this setting, frictional delivery during tracking through narrow or tortuous paths is both a nuisance and a source of off-target vascular and systemic delivery. These results encourage the development of technologies and procedures for coating protection during tracking at bends [49]. Currently, most DCBs, including those used in our experiments, are protected from premature drug loss during handling by a protective sheath, which is removed prior to device insertion and leaves the device unprotected during tracking. While coating on balloon folds is expected to be better protected from tracking loss than coating on fold ridges, even greater protection may be afforded by protective coating layers designed to expose coating only during inflation [50].

### 4.5. Distribution to Systemic Tissues

Sampling of systemic organ tissue revealed high paclitaxel concentrations in lung (277.2 ± 228.1 ng/g), kidney (92.8 ± 61.2 ng/g), and liver (35.3 ± 20.1 ng/g) tissue. A similar concentration distribution was previously reported for the IN.PACT DCB [40], 118 ng/g in lung, 4.2 ng/g in kidney, and 1.11 ng/g in liver. Our study design limits the ability to specify whether these concentrations derive from capturing systemic circulating particles released during tracking or from later detached or dissolved coating particles.

Given the slow dissolution kinetics of paclitaxel drug coatings, it can be assumed that coating particles released into plasma retain in their solid form during our short (<1 h) intervention time [4,5,7]. This in turn would suggest that the relative magnitude of paclitaxel distributions in sampled systemic organs would scale with the cardiac output to each of the organs, which is in qualitative concordance with our measurements. While 100% of the cardiac output is going through the lung, the kidneys receive 13% and liver tissue about 3-fold less (4.3%) cardiac output in swine [51]. This correlation indicates the potential fast distribution of particles lost during tracking within the blood stream and subsequent capture in small vasculature of systemic organs, which could explain the rapid drop in paclitaxel plasma levels between tracking and inflation. More rigorous support for this hypothesis is beyond the scope of the current study and would require additional sampling of systemic organs immediately post-tracking and post-inflation.

### 4.6. Limitations

The animal, benchtop, and computational models we employed each harbor limitations when compared to clinical reality.

Despite being widely accepted as a pre-clinical model for cardiovascular disease, ISR and naïve porcine arteries lack biological complexity compared to human pathology and might limit the translation of the here-reported tissue concentration data. While the precise values may not be representative of the clinical setting, there is reason to believe that the principles and forces underlying drug-based delivery from DCBs are similar in animals and humans. Additionally, guided catheter-assisted access through the common femoral artery is the most common practice for endovascular treatment of peripheral arterial disease in humans, but we used a shorter introducer sheath through the carotid artery. This variation resulted in a longer and more torturous unprotected tracking path in our in vivo experiment compared to standard clinical intervention. We chose this approach to analyze the effect of tracking loss in a “worst case” scenario, comparable with alterative access sites like brachial artery in challenging peripheral arterial disease cases [17], or tracking through the complex geometries typical for coronary artery treatments [52]. Despite the difference in tracking route with common clinical practice, the presented underlying mechanisms, leading to high levels of drug loss during tracking over bend and non-target frictional delivery, remain and should be taken into consideration when selecting access sites and tracking paths in human interventions.

We further did not evaluate the effect of premature drug loss on the DCB efficacy on overall disease treatment. While the biophysical forces governing drug and coating delivery are largely not drug-specific, the pharmacological consequences of drug distribution in various compartments are drug-specific and should be studied in dedicated long-term studies.

Additionally, our in vivo experiments did not control for the individual influence of hemodynamic factors like blood flow or mechanical stresses separately, and our benchtop experiments examined the effects of mechanical shear forces in the absence of hemodynamics. As we could not find any published data offering a direct correlation of hemodynamic forces on DCB drug loss, future studies will have to examine the importance of this limitation.

For both benchtop and computational models, a series of assumptions, approximations, and simplifications were made to enforce the feasibility of those experiments. For example, the contact pressure prediction during in silico tracking was simulated in a vessel wall as a discrete rigid shell body, which has been shown to be an accepted approximation [28].

Moreover, the present series of in vivo, in vitro, and in silico experiments were only performed for one specific DCB device. The current state of research shows significant variations in coating transfer, retention, and wash-out depending on the coating formulation, morphology, and other parameters, indicating that there is no general class effect for DCBs, which potentially compromises the transfer of our results onto other devices.

## 5. Conclusions

The presented synergistic animal, benchtop, and computational study elucidated premature tracking loss at vascular bends and its contributions to local and off-target drug delivery. Specifically, we showed that (a) tracking over a bend is the main contributor to systemic drug loss, suggesting the need to integrate post-tracking, pre-inflation plasma sampling into standard safety protocols, and that (b) even low-pressure frictional interactions of DCB at upstream vascular bends can efficiently transfer coating to underlying off-target arterial tissue, thereby limiting the available drug for inflation-driven delivery at the treatment site. Thus, it is incumbent upon us to develop procedural and technological innovations for protection of drug coating from frictional loss at vascular bends. In this regard, computational modeling of DCB–vessel interaction appears to hold promise.

## Data Availability

Data are contained within the article.

## Supplementary Materials

The following supporting information can be downloaded at https://www.mdpi.com/article/10.3390/pharmaceutics17020197/s1: Figure S1: Graphical representation of arterial vasculature-reconstruction based on 2D CT-scans. Figure S2: Graphical representation of 3D curvature distribution along pig vasculature. Figure S3: Left: Three point bending test of PearlFlow catheter. Right: Measured Youngs’s modulus of PearlFlow catheter for the three tested specimens. Figure S4: First the Young’s modulus of the full balloon catheter was calculated (A), then of a hollow cylinder (B) and finally the hollow cylinder of the balloon catheter including the guidewire (C). Figure S5: For the tracking simulation an 80 cm catheter/guidewire equivalent (top) was placed into the reconstructed arterial vasculature (bottom) and was released after horizontal and vertical translation. Figure S6: Geometry of in vitro tracking tubing. Figure S7: Pressure distribution of stamping simulation averaging at 0.11 atm (left) and 0.16 atm (right). Table S1: Comparison of paclitaxel concentration with literature values [10,18,21,22,23,28,29,30,38,39,40,41,53,54,55,56,57].
