# Supplementary material for: Drug Loss at Arterial Bends Can Dominate Off-Target Drug Delivery by Paclitaxel-Coated Balloons
Source: Pharmaceutics. 2025 Feb 4;17(2):197. doi: 10.3390/pharmaceutics17020197 (PMC11858837; doi:10.3390/pharmaceutics17020197)
Supplement: Supplementary file 1 [file pharmaceutics-17-00197-s001.zip › pharmaceutics-3396495-supplementary.pdf]

Supplementary Material for "Drug Loss at Arterial Bends Can Dominate Off-Target Drug Delivery by Paclitaxel-Coated Balloons"

Linnea Tscheuschner[1]*, Efstathios Stratakos[2], Marios Kostakis[3], Miltiadis Gravanis[4], Michalis Katsimpoulas [5], Giancarlo Pennati [2], Fragiska Sigala[1] and Abraham R. Tzafriri[6]

Concept

The dynamic frictional forces exerted on the drug-coated balloon (DCB) during in vivo and ex vivo tracking were the product of a dynamic friction coefficient and contact normal force. Given that the dynamic friction coefficient remains relatively constant even when the tissue is excised, our objective was to calculate the normal forces required to reproduce the in vivo frictional forces generated during horizontal displacement. The assumptions underlying DCB–vessel mechanical interactions are detailed in subsequent sections.

Artery model

For the reconstruction of the arterial vasculature, 2D CT scans from [29] were utilized as a background in SpaceClaim to reconstruct the 3D centerline of the vessels (Fig. 1). Circular cross-sections, based on the luminal diameter reported in [53], were created at specific locations along the vasculature and connected using the surface blend tool, guided by the centerline. The radii of the arcs at regions of maximum curvature were measured (Fig. 2).

The reconstructed pig vasculature model was then imported into Abaqus CAE (Dassault Systems, Paris, France) as a discrete rigid shell body. This assumption aligns with the recent literature indicating that modeling the lumen as a rigid body is as accurate as using deformable vessel walls for assessing interfacial contact pressure during tracking [28]. The model was meshed with a total of 22,617 elements, comprising 22,488 4-node 3-D bilinear rigid quadrilateral elements and 129 3-node linear triangular elements, and included 22,574 nodes.

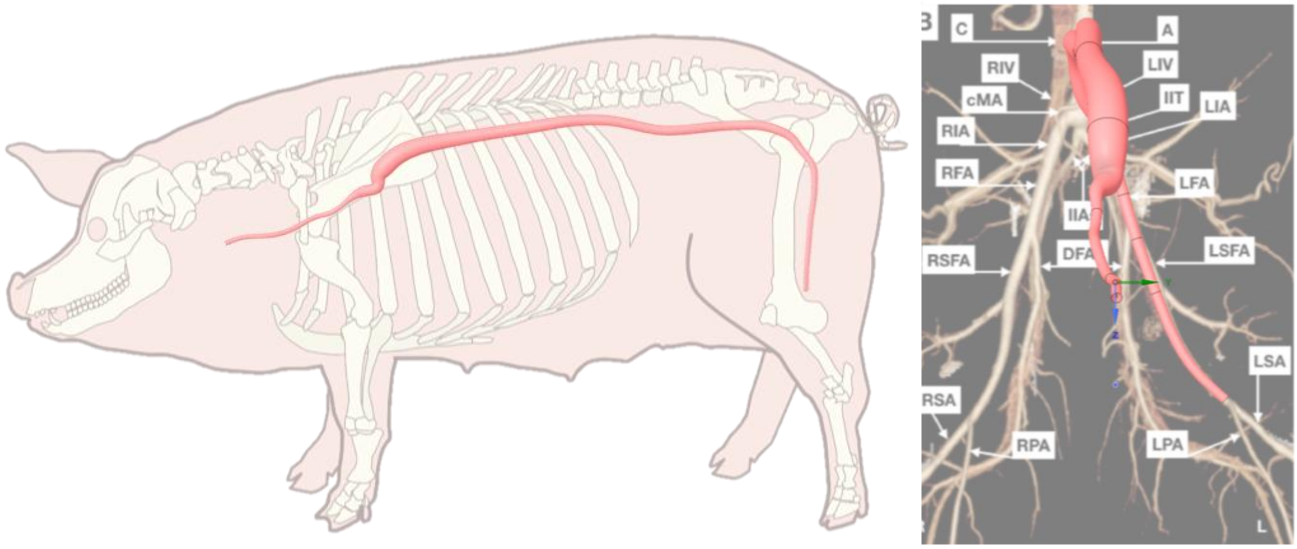

Figure 1. Graphical representation of arterial vasculature—reconstruction based on 2D CT-scans.

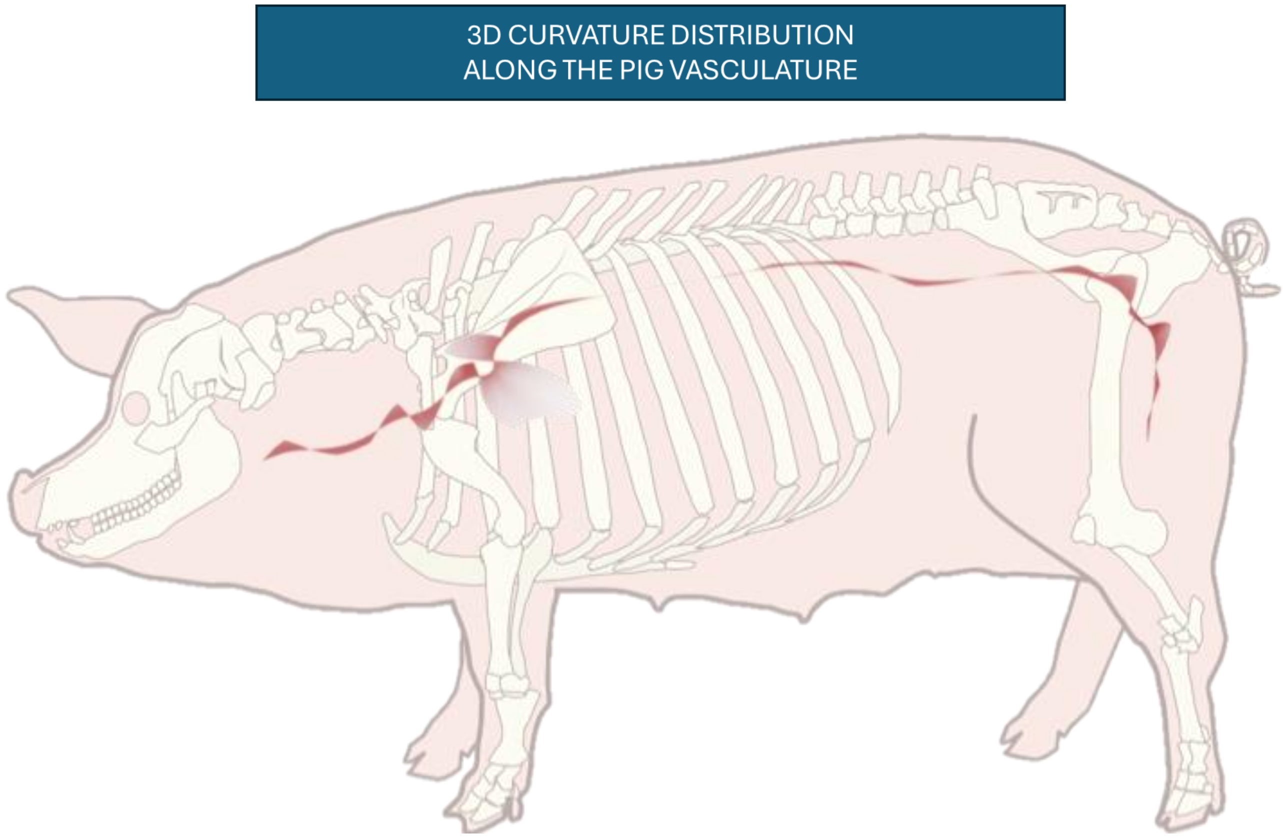

Figure 2. Graphical representation of 3D curvature distribution along pig vasculature.

Catheter–guidewire model

Geometry

The catheter was simulated as an 80 cm long beam.

Material

To characterize the material properties of the DCB catheter, 3-point bending tests were performed using three 8 mm long segments of the catheter (Fig. 3). The tests were conducted in a quasi-static manner with a displacement rate of 2.5 μm/s. Force–displacement data were recorded using a 5 N load cell. The median Young's modulus for a full cylindrical catheter was calculated based on the following analytical equations (Fig. 4):

Polar Moment of Inertia (I):

$$I = \frac{\pi \cdot R^4}{4}$$

Young's Modulus of the catheter (E):

$$E = \frac{48 \cdot \delta \cdot I}{F \cdot L} = 495.63 \text{ MPa}$$

where:
R is the radius of the catheter,
I is the polar moment of inertia,
E is the material Young's modulus,
F is the applied normal experimental force,
L is the length between the external supports (pins),
δ is the experimental displacement.

Given that the catheter is a hollow cylinder, we adjusted our calculation to account for the internal diameter (Fig. 4). The internal diameter was measured experimentally, and the Young's modulus for the hollow cylinder was computed using the following equation:

Young's Modulus for a Hollow Cylinder (E_2):

$$E_2 = \frac{32 \cdot \pi^2 \cdot E \cdot ({R_{ext}}^4 - {R_{int}}^4)}{{R_{ext}}^4} = 504.89 \text{ MPa}$$

where:

R_{ext} is the external radius of the catheter,

R_{int} is the internal radius of the catheter,

To determine the equivalent stiffness of the catheter combined with the guidewire (Fig. 4), the guidewire's material properties were obtained from the literature [54], and its diameter was assumed to fully occupy the catheter's internal hole. The total equivalent Young’s modulus E_{tot} of the catheter and guidewire system was calculated using:

Total Equivalent Young’s Modulus (E_{tot}):

$$\frac{E \cdot \frac{\pi}{32} \cdot ({R_{ext}}^4 - {R_{int}}^4) + E_{guidwire} \cdot \frac{\pi}{32} \cdot {R_{int}}^4}{\frac{\pi}{32} \cdot {R_{ext}}^4} = 1412.61 \text{ MPa}$$

where:

$E_{guidewire}$ is the Young’s modulus of the guidewire.

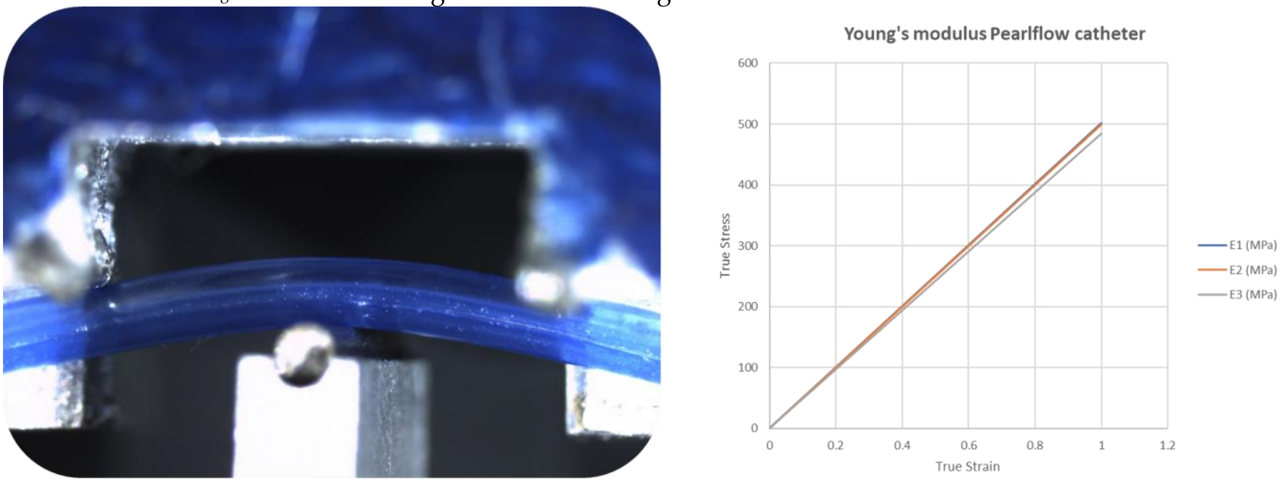

Figure 3. Left: Three-point bending test of PearlFlow catheter. Right: Measured Youngs’s modulus of PearlFlow catheter for the three tested specimens.

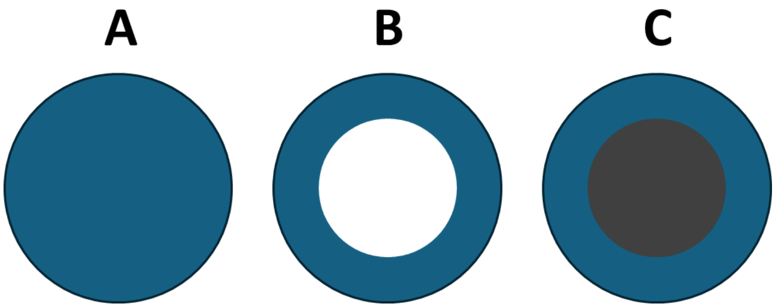

Figure 4. First, the Young’s modulus of the full balloon catheter was calculated (A), then of a hollow cylinder (B), and, finally, the hollow cylinder of the balloon catheter including the guidewire (C).

Mesh

The model was discretized with a total of 401 nodes and 400 elements, where 400 linear line elements of type B31 were employed. The B31 element represents a 2-node linear beam in space, suitable for capturing linear structural behavior.

Simulation steps

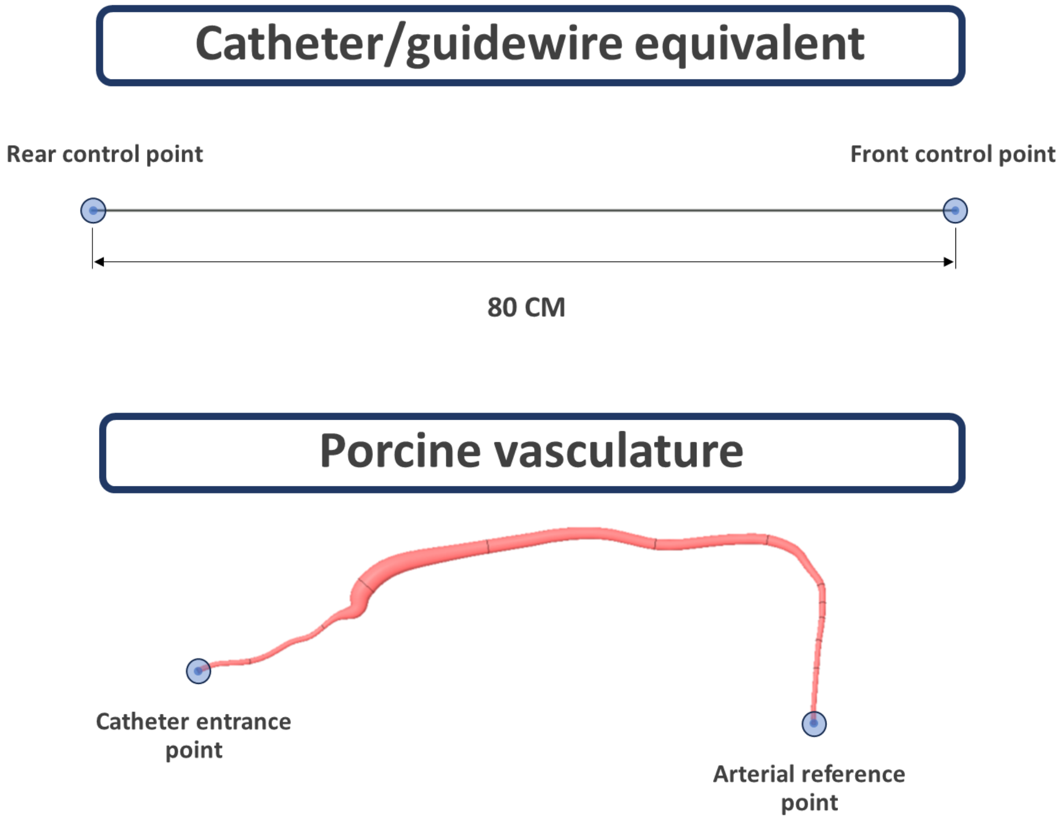

Figure 5. For the tracking simulation, an 80 cm catheter–guidewire equivalent (top) was placed into the reconstructed arterial vasculature (bottom) and was released after horizontal and vertical translation.

Simulation Step 1: Horizontal Translation

The first simulation step involved a horizontal translation of the front node, with a total duration of 0.5 seconds. Boundary conditions (BCs) included a horizontal displacement of 550 mm applied to the front control point (node). The rear control point (node) was constrained, prohibiting movement along the y and z planes, ensuring stability in these directions. The artery was fixed through a Reference Point (RP) to prevent any movement.

Simulation Step 2: Vertical Translation

Following the horizontal translation, the second step consisted of a vertical translation applied to the front control point along the z-axis, also for a duration of 0.5 seconds. The front node was displaced by 170 mm vertically. Similar to Step 1, the rear control point was constrained from moving on the y and z planes, and the artery was maintained in a fixed position through the RP.

Simulation Step 3: Relaxation

In the final step, the system underwent a relaxation phase lasting 2 seconds. During this period, the artery remained fixed through the RP, allowing for the structure to settle and reach an equilibrium state following the applied translations (Fig. 5).

Contact

The contact algorithm used for the tracking simulation incorporated hard contact to model the normal interaction between the guidewire–catheter and the vessel endolumen. PVC tubing has a dynamic friction coefficient of 0.23 [55], while the catheter–artery coefficient of friction is reported to vary between 0.015 and 0.13, depending on the angle [56]. For the tangential interaction, a friction coefficient with a penalty parameter of 0.2 was applied [18].

In silico tracking through tubing

For the tracking simulation within the tube, the same modeling approach used in the tracking simulation of pig vasculature was implemented. The primary difference in this case was the application of a 414 mm vertical displacement during the first simulation step, followed by a 230 mm horizontal displacement. The geometry of the tube, as described in the main text, is illustrated in the following figure (Fig. 6).

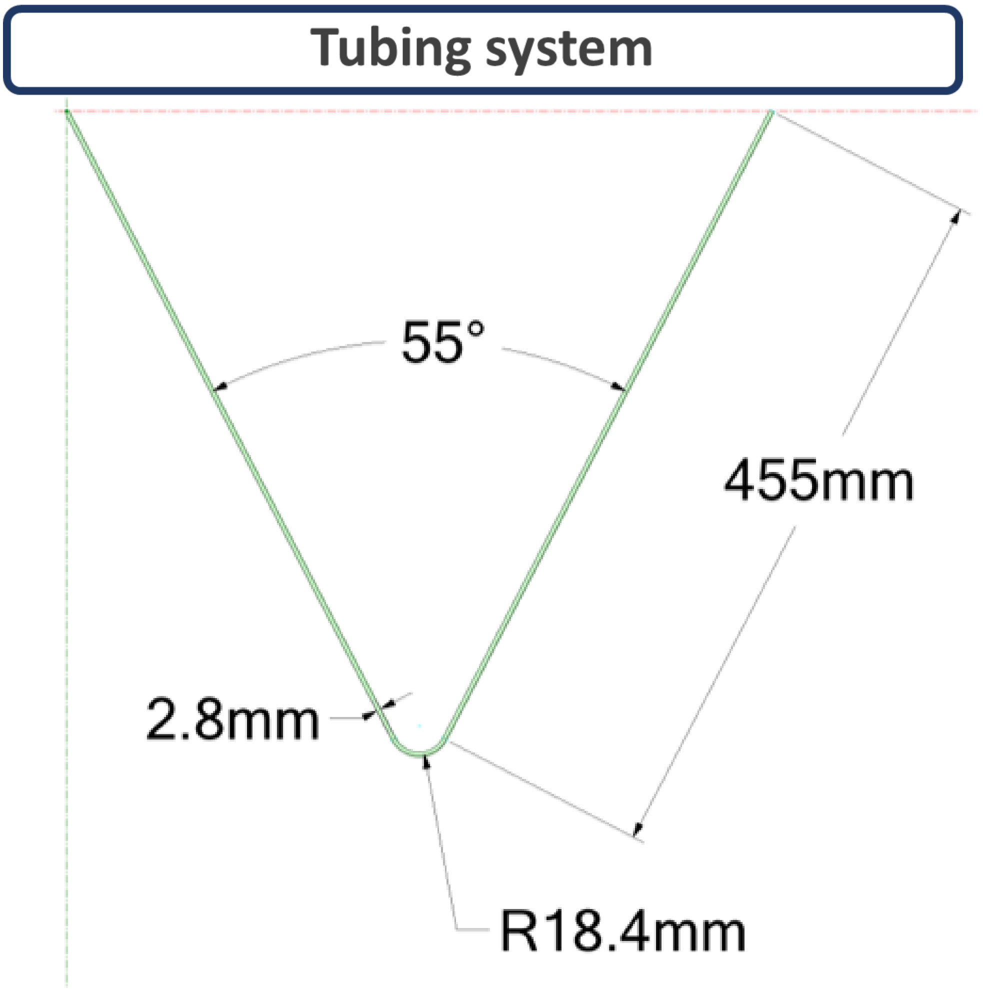

Figure 6. Geometry of in vitro tracking tubing.

Stamping simulation—pressure distribution

Static compression simulations (Fig. 7) presented in Stratakos et al. [10] illustrate the pressure distribution on the endoluminal surface of the tissue during stamping experiments. By averaging the contact pressure in regions where the coating was present on the physical specimens, we observed mean pressures of 0.11 atm and 0.16 atm for the two frictional force conditions replicated in this study.

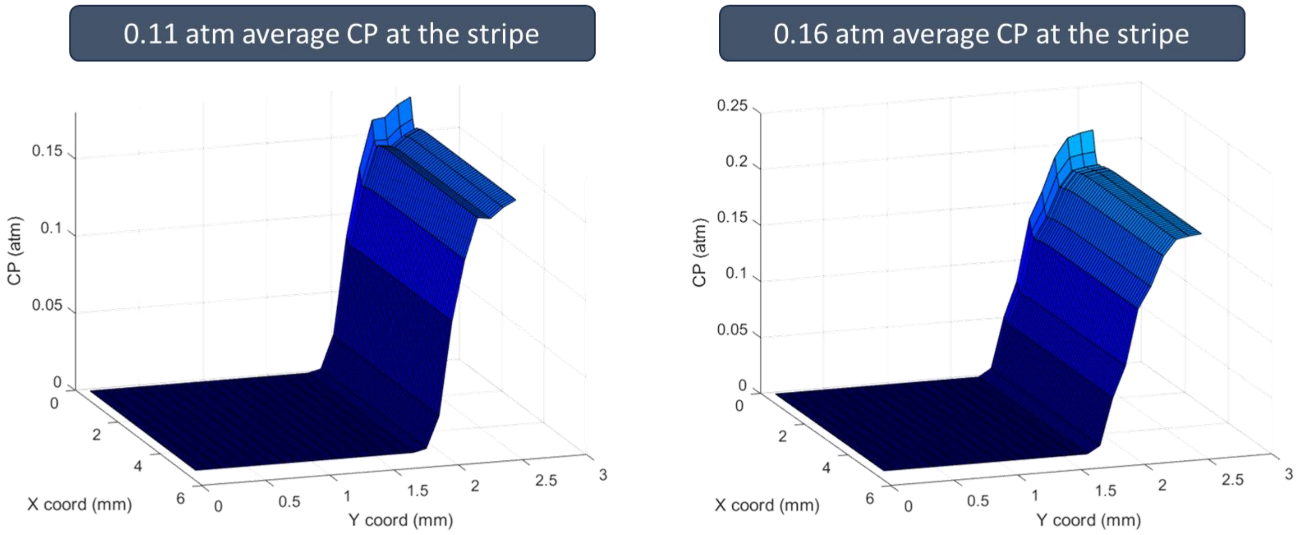

Figure 7. Pressure distribution of stamping simulation averaging at 0.11 atm (left) and 0.16 atm (right).

Table S1. Comparison of paclitaxel concentration with literature values.

Sampling	Time point/ location	Device	Reference	Animal	Value
Plasma	Post-tracking	PearlFlow (3 µg/mm²)	Present study	swine	746.9 ± 336.3 ng/mL
	Immediately after inflation	PearlFlow (3 µg/mm²)	Present study	swine	24.6 ± 20.2 ng/mL
	3 min post inflation	Lutonix (2 µg/mm²)	[23]	swine	2.88 ± 0.74 ng/mL
	1 min post inflation	Passeo (3 µg/mm²)	[38]	swine	301 ± 90 ng/mL
	5 min post inflation			swine	200 ng/mL
	Immediately after inflation	Stellarex (2 µg/mm²)	[41]	human	54.4 ± 116.8 ng/mL
	Immediately after inflation	Pantera Lux (3 µg/mm²)	[39]	rabbit	0.3 ± 0.17 µg/mL
	Immediately after inflation	IN.PACT (3.5 µg/mm²)	[40]	swine	1.6 ng/mL
	Immediately after inflation	Lutonix (2 µg/mm²)	[57]	human	58.4 ± 83.2 ng/mL
Target Vessel	˜1h post inflation	PearlFlow (3 µg/mm²)	Present study	swine	79.84 ± 119.27 ng/mg (healthy)
	1h post inflation	Lutonix (2 µg/mm²)	[23]	swine	58.8 ± 54.2 ng/mg
	30 min post inflation	Passeo (3 µg/mm²)	[38]	swine	30 ng/mg (mean for 28 days 15.68 ±16.33 ng/mg)
	1 h post inflation	Pantera Lux (3 µg/mm²)	[39]	rabbit	168.54 ± 83.48 ng/mg (healthy) 623.05 ± 125.75 ng/mg (diseased)
	1 h post inflation	Kanshas (3.2 µg/mm²)	[22]	swine	813 ng/mg
	Immediately after inflation	IN.PACT (3.5 µg/mm²)	[40]	swine	35.43 ng/mg
Downstream skeletal tissue	˜1h post inflation	PearlFlow (3 µg/mm²)	Present study	swine	Min 0.009 ± 0.005 ng/mg Max 0.089 ± 0.087 ng/g
	1 h post inflation	Kanshas (3.2 µg/mm²)	[22]	swine	1.15 ± 0.34 ng/mg
	28 days post inflation	3x IN.PACT (3.5 µg/mm²)	[58]	swine	0.23 ± 0.15 ng/mg
	28 days post inflation	3x Ranger (2 µg/mm²)	[58]	swine	0.08 ± 0.07 ng/mg
	28 days post inflation	3x Stellarex (2 µg/mm²)	[58]	swine	0.11 ± 0.12 ng/mg

	Immediately after inflation	IN.PACT (3.5 μg/mm²)	[40]	swine	1.2 ng/mg
	28 days post inflation	Lutonix 2 μg/mm²)	[59]	swine	0.001 ± 0.0007 ng/mg
	28 days post inflation	IN.PACT (3.5 μg/mm²)	[59]	swine	0.07± 0.09 ng/mg
Upstream Tissue	~1h post inflation	PearlFlow (3 μg/mm²)	Present study	swine	4.35 ± 4.08 ng/g
Systemic organs	~1h post inflation	PearlFlow (3 μg/mm²)	Present study	swine	0.28 ± 0.23 ng/mg (lung) 0.04 ± 0.02 ng/mg (liver) 0.09 ± 0.06 ng/mg (kidney)
	Immediately after inflation	IN.PACT (3.5 μg/mm²)	[40]	swine	1.18 ng/mg (lung) 0.011 ng/mg (liver) 0.042 ng/mg (kidney)

References

10. Stratakos, E.; Tscheuschner, L.; Vincenzi, L.; Pedrinazzi, E.; Sigala, F.; D'Andrea, L.; Gastaldi, D.; Berti, F.; Tzafriri, A.R.; Pennati, G. A Novel In Silico - Ex Vivo Model for Correlating Coating Transfer to Tissue with Local Drug-Coated Balloon-Vessel Contact Pressures. *Ann Biomed Eng* **2024**, doi:10.1007/s10439-024-03634-6.

18. Stratakos, E.; Antonini, L.; Poletti, G.; Berti, F.; Tzafriri, A.R.; Petrini, L.; Pennati, G. Investigating Balloon-Vessel Contact Pressure Patterns in Angioplasty: In Silico Insights for Drug-Coated Balloons. *Ann Biomed Eng* **2023**, *51*, 2908–2922, doi:10.1007/S10439-023-03359-Y/FIGURES/8.

22. Sato, Y.; Kawakami, R.; Kawai, K.; Konishi, T.; Vozenilek, A.E.; Ghosh, S.K.B.; Abebe, B.; Romero, M.E.; Kolodgie, F.D.; Virmani, R.; et al. Local, Downstream, and Systemic Evaluation after Femoral Artery Angioplasty with Kanshas Drug-Coated Balloons In Vitro and in a Healthy Swine Model. *Journal of Vascular and Interventional Radiology* **2023**, *34*, 1166-1175.e2, doi:10.1016/j.jvir.2023.03.024.

23. Yazdani, S.K.; Pacheco, E.; Nakano, M.; Otsuka, F.; Naisbitt, S.; Kolodgie, F.D.; Ladich, E.; Rousselle, S.; Virmani, R. Vascular, Downstream, and Pharmacokinetic Responses to Treatment with a Low Dose Drug-Coated Balloon in a Swine Femoral Artery Model. *Catheterization and Cardiovascular Interventions* **2014**, *83*, 132–140, doi:10.1002/CCD.24995.

28. Symes, D.G.; McNamara, L.M.; Conway, C. Computational Investigation of Vessel Injury Due to Catheter Tracking During Transcatheter Aortic Valve Replacement. *Ann Biomed Eng* **2024**, *52*, 1554–1567, doi:10.1007/S10439-024-03462-8/FIGURES/8.

29. Edwards, J.; Abdou, H.; Patel, N.; Madurska, M.J.; Poe, K.; Bonin, J.E.; Richmond, M.J.; Rasmussen, T.E.; Morrison, J.J. The Functional Vascular Anatomy of the Swine for Research. *Vascular* **2022**, *30*, 392–402, doi:10.1177/1708538121996500/ASSET/IMAGES/LARGE/10.1177_1708538121996500-FIG12.JPEG.

38. Abadal, J.M.; Vazquez, E.; Morales, M.; Toro, A.; Quintana, M.; Araujo, M. Pharmacokinetic Evaluation of Two Paclitaxel-Coated Balloons with Different Drug Load in a Short-Term Porcine Study. *Cardiovasc Intervent Radiol* **2016**, *39*, 1152–1158, doi:10.1007/S00270-016-1346-X/FIGURES/4.

39. Fernández-Parra, R.; Laborda, A.; Lahuerta, C.; Lostalé, F.; Aramayona, J.; De Blas, I.; De Gregorio, M.A. Pharmacokinetic Study of Paclitaxel Concentration after Drug-Eluting Balloon Angioplasty in the Iliac Artery of Healthy and Atherosclerotic Rabbit Models. *J Vasc Interv Radiol* **2015**, *26*, 1380-1387.e1, doi:10.1016/J.JVIR.2015.05.022.
40. Tunev, S. IN.PACT Technology and Pre-Clinical Evidence 2024.
41. Krishnan, P.; Faries, P.; Niazi, K.; Jain, A.; Sachar, R.; Bachinsky, W.B.; Cardenas, J.; Werner, M.; Brodmann, M.; Mustapha, J.A.; et al. Stellarex Drug-Coated Balloon for Treatment of Femoropopliteal Disease: Twelve-Month Outcomes From the Randomized ILLUMENATE Pivotal and Pharmacokinetic Studies. *Circulation* **2017**, *136*, 1102–1113, doi:10.1161/CIRCULATIONAHA.117.028893.
53. Smit, J.H.A.; Leonardi, E.P.; Chaves, R.H. de F.; Furlaneto, I.P.; da Silva, C.M.S.; Abib, S. de C.V.; Góes Junior, A.M. de O. Image-Guided Study of Swine Anatomy as a Tool for Urologic Surgery and Training. *Acta Cir Bras* **2020**, *35*, 1–11, doi:10.1590/ACB351208.
54. Berti, F.; Bridio, S.; Luraghi, G.; Pant, S.; Allegretti, D.; Pennati, G.; Petrini, L. Reliable Numerical Models of Nickel-Titanium Stents: How to Deduce the Specific Material Properties from Testing Real Devices. *Ann Biomed Eng* **2022**, *50*, 467, doi:10.1007/S10439-022-02932-1.
55. Akbar, F.; Rizkiyah, N.; Abdullah, M. Minimal Tools for Accurately Measuring the Coefficient of Kinetic Friction 2019.
56. Takashima, K.; Shimomura, R.; Kitou, T.; Terada, H.; Yoshinaka, K.; Ikeuchi, K. Contact and Friction between Catheter and Blood Vessel. *Tribol Int* **2007**, *40*, 319–328, doi:10.1016/J.TRIBOINT.2005.10.010.
57. Scheinert, D.; Duda, S.; Zeller, T.; Krankenberg, H.; Ricke, J.; Bosiers, M.; Tepe, G.; Naisbitt, S.; Rosenfield, K. The LEVANT i (Lutonix Paclitaxel-Coated Balloon for the Prevention of Femoropopliteal Restenosis) Trial for Femoropopliteal Revascularization: First-in-Human Randomized Trial of Low-Dose Drug-Coated Balloon versus Uncoated Balloon Angioplasty. *JACC Cardiovasc Interv* **2014**, *7*, 10–19, doi:10.1016/J.JCIN.2013.05.022.
58. Torii, S.; Jinnouchi, H.; Sakamoto, A.; Romero, M.E.; Kolodgie, F.D.; Virmani, R.; Finn, A. V. Comparison of Biologic Effect and Particulate Embolization after Femoral Artery Treatment with Three Drug-Coated Balloons in Healthy Swine Model. *Journal of Vascular and Interventional Radiology* **2019**, *30*, 103–109, doi:10.1016/J.JVIR.2018.07.025.
59. Kolodgie, F.D.; Pacheco, E.; Yahagi, K.; Mori, H.; Ladich, E.; Virmani, R. Comparison of Particulate Embolization after Femoral Artery Treatment with IN.PACT Admiral versus Lutonix 035 Paclitaxel-Coated Balloons in Healthy Swine. *J Vasc Interv Radiol* **2016**, *27*, 1676-1685.e2, doi:10.1016/J.JVIR.2016.06.036.
